# Engineering robust microorganisms for organic acid production

**DOI:** 10.1093/jimb/kuab067

**Published:** 2021-09-21

**Authors:** Vinh G Tran, Huimin Zhao

**Affiliations:** Department of Chemical and Biomolecular Engineering, U.S. Department of Energy Center for Bioenergy and Bioproducts Innovation (CABBI), Carl R. Woese Institute for Genomic Biology, University of Illinois at Urbana-Champaign, Urbana, IL 61801, USA; Department of Chemical and Biomolecular Engineering, U.S. Department of Energy Center for Bioenergy and Bioproducts Innovation (CABBI), Carl R. Woese Institute for Genomic Biology, University of Illinois at Urbana-Champaign, Urbana, IL 61801, USA; Departments of Chemistry, Biochemistry, and Bioengineering, University of Illinois at Urbana-Champaign, Urbana, IL 61801, USA

**Keywords:** Organic acids, Robustness, Low pH, Acid stress, Lignocellulosic inhibitors

## Abstract

Organic acids are an important class of compounds that can be produced by microbial conversion of renewable feedstocks and have huge demands and broad applications in food, chemical, and pharmaceutical industries. An economically viable fermentation process for production of organic acids requires robust microbial cell factories with excellent tolerance to low pH conditions, high concentrations of organic acids, and lignocellulosic inhibitors. In this review, we summarize various strategies to engineer robust microorganisms for organic acid production and highlight their applications in a few recent examples.

## Introduction

Global concerns pertaining to the unsustainable chemical productions using fossil resources and the consequential environmental impacts have motivated the development of microbial cell factories for production of fuels and chemicals using renewable biomass (Du et al., 2011; Lian et al., 2018b). Organic acids are important value-added products that have diverse applications in food, pharmaceutical, and chemical industries, and metabolic engineering and synthetic biology have been extensively employed to engineer platform strains for organic acid production (Liu et al., 2017). However, microbial production of organic acids suffers from some challenges. Bacteria are typically not tolerant to low pH conditions. In particular, gram-negative bacteria, such as *Escherichia coli*, have the periplasm encased by the outer and inner membranes. The outer membrane harbors nonspecific transporters, such as porins, and protons can freely diffuse through these transporters (Hong et al., 2012). Since the periplasm houses essential enzymes and transporters for the uptake of nutrient molecules (Nikaido, 2003), the acidification of the periplasm resulted from the accumulation of protons can denature the periplasmic proteins and disrupt their functions (Hong et al., 2012), leading to reduced cell growth. Thus, while the production of organic acids using bacteria can accomplish very high titers and yields, the inhibitory effects of low pH on bacterial growth necessitate the use of neutralization agents such as calcium carbonate to maintain fermentations at neutral pH, which is higher than the pKa values of all organic acids (e.g., 4.21 for succinic acid; 3.86 for lactic acid) (Yin et al., 2015). Subsequently, the salt forms of organic acids are obtained. An acidification step using strong acid (e.g., sulfuric acid) is thus necessary to recover the undissociated forms of organic acids but leads to the formation of gypsum, rendering downstream processing costly.

On the other hand, although the titers and yields of organic acids attained by yeasts are lower than those obtained from bacteria, yeasts can tolerate much lower pH conditions since their plasma membranes are not permeable to protons. However, at low pH, organic acids are in their undissociated forms and can cross the yeast cell membrane freely by diffusion at high concentrations (Casal et al., 2008). Upon entering the cytoplasm, the organic acids will dissociate into protons and their anionic forms, leading to cytosolic acidification. Accumulation of organic acid anions can also be toxic to the yeasts. Moreover, when lignocellulosic biomass is used as a feedstock, high tolerance of microorganisms to inhibitors present in lignocellulosic hydrolysates is another important prerequisite (Jin & Cate, 2017). Lignocellulosic inhibitors, including phenolic molecules and furans, are the by-products derived from the pretreatment of lignocellulose (Wang et al., 2018b). Phenolic compounds, such as vanillic acid and catechol, can penetrate and damage the cell membrane, leading to the leakage of intracellular components. Furan aldehydes, including furfural and 5-(hydroxymethyl)-2-furaldehyde (HMF), can inhibit glycolytic genes. Furthermore, lignocellulosic inhibitors can increase the level of reactive oxygen species, damaging the cytoskeleton and mitochondria.

Thus, for efficient and economical microbial production of organic acids, the platform organisms should be able to tolerate highly acidic conditions, high concentrations of organic acids, and stress from lignocellulosic inhibitors. In this review, we first introduce some strategies frequently utilized to improve the robustness of various microbial hosts for organic acid production. The strategies include transcriptome analysis-guided engineering, transcription machinery engineering, adaptive laboratory evolution (ALE), and genome-scale engineering. We then highlight their successful applications in a number of recent case studies.

## Commonly Used Strategies to Improve Host Robustness

**Fig. 1.**
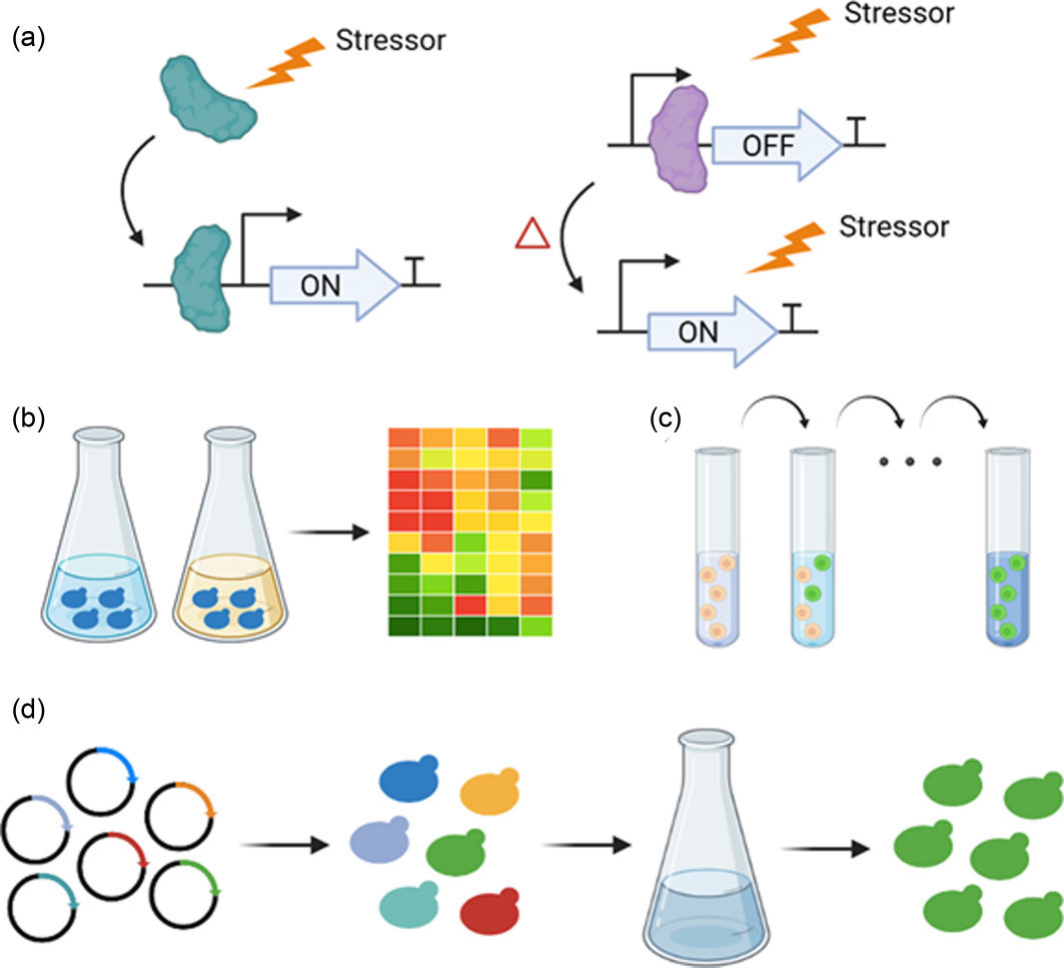
Commonly utilized strategies for enhancing host robustness. (a) Overexpression or deletion of transcription factors associated with stress tolerance. (b) Transcriptome analysis-guided identification of rational targets that can confer enhanced stress tolerance. (c) Adaptive laboratory evolution by serial transfer for improved tolerance to stress. (d) Transformation of pooled library and enrichment of genetic perturbations relevant to stress tolerance.

The robustness of microorganisms toward low pH, acid stress, and lignocellulosic inhibitors can be enhanced by several strategies (Fig. 1). Transcriptome analysis can identify the dynamic changes in gene expression induced by stresses (Horinouchi et al., 2018). Microbes are grown in the presence and absence of stresses, and transcription profiling can then be employed to compare the discrepancies in gene expression levels between the two conditions, leading to the identification of rational targets that can possibly confer enhanced tolerance to stresses (Fig. 1B). Transcription factors can sense small molecules and stimuli from internal and external environments; manipulation of transcription factors associated with stress response by overexpression or deletion can alter the expression levels of their target genes, which can potentially result in an improved tolerance phenotype (Fig. 1A) (Wan et al., 2019). Furthermore, ALE is an indispensable tool in metabolic engineering for strain development and has been highly effective in improving microbial tolerance to stress, such as high salinity, high temperature, and low pH (Sandberg et al., 2019). Microorganisms are cultivated in defined environments and serially transferred to harsher conditions for extended periods of time, allowing the natural selection of improved phenotypes (Fig. 1C). Finally, genome-scale engineering can efficiently create genetic variations throughout the whole genome in parallel and has been successfully applied to improve chemical productions, substrate utilization, and tolerance to toxic compounds (Cao et al., 2020b; Si et al., 2015b). Pooled libraries based on modulation strategies such as gene deletion or interference are constructed and transformed into microorganisms (Fig. 1D). Selective pressure such as high concentration of lignocellulosic inhibitors is then applied, leading to the enrichment of genetic perturbations relevant to the phenotype of interest.

## Engineering for Tolerance to Low pH

### Transcriptome Analysis-Guided Engineering

Transcriptional profiling was performed on the *Lactococcus lactis* strains experiencing acid shock at pH 4, revealing the upregulations in response to low pH stress, one of which was the overexpression of the ATP-binding cassette (ABC) transporters (Zhu et al., 2019). Reverse engineering showed that strains expressing the ABC transporters RbsA and RbsB (ribose import ATP-binding proteins), MsmK (ATPase), and DppA (dipeptide permease) exhibited at least fivefold higher survival rates after exposure to acidic conditions at pH 4 for 3 h. Genes related to cold-shock proteins, fatty acid synthesis, and ATP generation were also found to be upregulated. In another study, comparative transcriptomics analysis allowed the comparisons of global gene expression in *Pichia kudriavzevii* at pH 2.0 and 5.5 (Ji et al., 2020). Genes related to arginine metabolism, such as arginine permease and arginase, were found to be highly upregulated under the low pH condition, suggesting that arginine metabolism might play an important role in the tolerance of *P. kudriavzevii* to highly acidic conditions. Supplementation of extracellular arginine or overexpression of genes involved in arginine biosynthesis, such as the arginine biosynthesis bifunctional protein ARGJ, was found to enhance the cell growth and maintain a neutral intracellular pH.

### Transcription Machinery Engineering

**Fig. 2.**
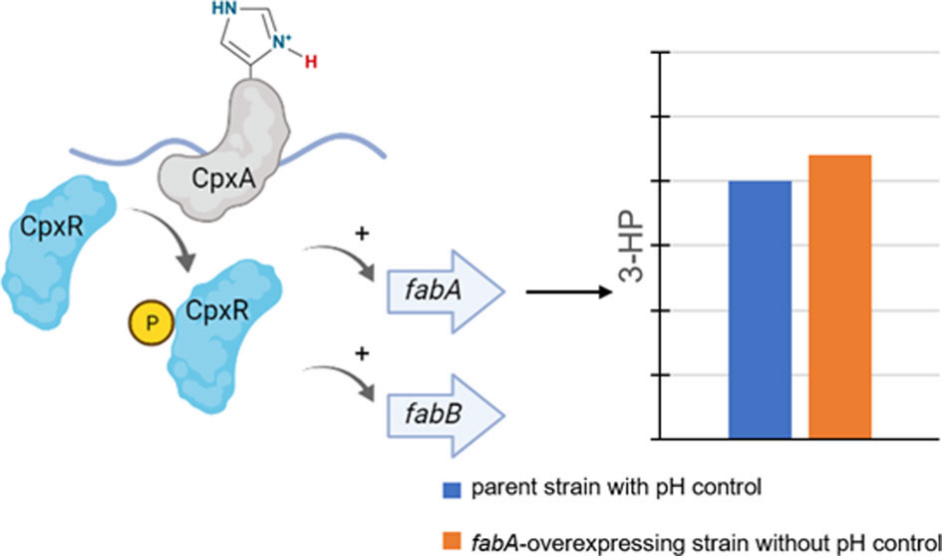
Scheme for the mechanism of two-component signal transduction system CpxRA (Xu et al., 2020). *CpxA* can sense acidic condition through protonation of histidine residues, leading to phosphorylation of CpxR and upregulation of *fabA* and *fabB* for synthesis of unsaturated fatty acids. Overexpression of *fabA* enabled similar 3-hydroxypropionic acid production with and without pH adjustment.

Tolerance to acidic condition at pH 2 by *Candida glabrata* was found to be linked to *RDS2*, a transcriptional regulator of glycerophospholipid metabolism (Wu et al., 2020). *RDS2* deletion reduced cell growth at pH 2, whereas overexpression of *RDS2* was beneficial in improving the cell survival at pH 2 by 17%, ATP content by 42%, and membrane permeability by 19% compared to the wild-type strain. In another study, an acid-tolerant response system required for *E. coli* growth at pH 4.2 was characterized (Xu et al., 2020). A two-component signal transduction system CpxRA was determined to sense acidic conditions through protonation of CpxA (sensor histidine kinase) periplasmic histidine residues, leading to phosphorylation of the transcriptional regulatory protein CpxR and upregulation of the *fabA* (3-hydroxyacyl-ACP dehydratase/isomerase) and *fabB* (β-ketoacyl-ACP synthase) for the synthesis of unsaturated fatty acids (Fig. 2). Alterations in lipid composition then decreased membrane fluidity and improved intracellular pH homeostasis. Overexpression of the *fabA* gene was also found to improve *E. coli* tolerance to acidic environments, enabling comparable 3-hydroxypropionic acid production with and without pH control (Xu et al., 2020).

### Adaptive Laboratory Evolution

*Actinobacillus succinogenes* is a potential microorganism for succinic acid production because of its ability to consume several substrates and strong growth rate under anaerobic conditions (Zhang et al., 2020). The wild-type *A. succinogenes* could produce 18.97 g/l of succinic acid with a yield of 0.68 g/g glucose at pH of 6.8. Nevertheless, at pH 5.8, the succinic acid titer was significantly decreased to 7.03 g/l, and no growth was observed when pH was 5.6. To improve the production at lower pH, ALE was performed over 1140 h by serial dilution in media with the pH ranging from 6.8 to 5.8. The best mutant exhibited a 2.36-fold increase in cell growth rate, and batch fermentation gave 20.77 g/l of succinic acid at pH 5.8, which was 2.95-fold higher than the wild-type strain. This mutant also had a higher intracellular ATP supply than the wild-type strain, which might contribute to the enhanced tolerance toward low pH stress.

In addition, *Saccharomyces cerevisiae* was evolved to acquire tolerance to HCl and lactic acid at pH 2.8 (Fletcher et al., 2017). The evolved strains adapted to HCl and lactic acid had 31.3% and 200% increase in maximum specific growth rates compared to the parent strain, respectively. For tolerance to HCl, genes related to iron transport, such as *FRE1* (ferric reductase), were disrupted to limit the uptake of iron, which becomes more toxic at lower pH; genes involved in sterol and lipid metabolism were also found to be upregulated. For tolerance to lactic acid, formation of a multicellular morphology and rapid lactate degradation by upregulation of CYB2, which converts lactate to pyruvate, were observed.

ALE was performed on *E. coli* for improved growth rate at pH 5.5 (Du et al., 2020). The evolved strains were able to grow 18% faster than the parent strain at pH 5.5 and had similar growth rates to the starting strain at neutral pH. RNA-seq revealed that genes related to amino acid metabolism and energy production were highly upregulated to combat the low pH stress. Deletion in *rpoC* encoding a subunit of RNA polymerase was observed in most of the evolved clones, and the authors speculated this mutation, rather than improving acid resistance, enabled faster growth rate under low pH condition.

### Genome-Scale Engineering

A genomic DNA library of the multi-stress-tolerant *Issatchenkia orientalis* was transformed into *S. cerevisiae*, and transformants were plated onto synthetic complete dextrose plates containing medium adjusted to pH 2.0 (Matsushika et al., 2016). A single colony appeared after 20 days of incubation, and the plasmid harbored in this colony was found to contain two open reading frames, one of which had high similarity with *S. cerevisiae GAS1* (β-1,3-glucanosyltransferase), a gene involved in the cell wall integrity pathway. Overexpression of this gene in wild-type *S. cerevisiae* enabled robust growth and higher ethanol production at pH 2 in the presence of 5% Na_2_SO_4_.

## Engineering for Tolerance to Acid Stress

### Transcriptome Analysis-Guided Engineering

Transcriptome analysis was performed to compare the differences in gene expression in ethanol fermentation using *S. cerevisiae* under acetic acid stress with or without zinc sulfate supplementation (Zhang et al., 2017). Downregulation in the transmembrane transporters for carboxylic acids, including an acetate transporter *ADY2*, was observed. Deletion of *ADY2* improved the tolerance of *S. cerevisiae* toward acetic acid and hydrogen peroxide stresses. This knockout also enhanced the ethanol production by 14.7% in the presence of 3.6 g/l of acetic acid. In another study, around 500 different *S. cerevisiae* strains were examined for tolerance toward acetic acid, and *S. cerevisiae* ATCC 38555 was observed to have a significantly higher growth rate in medium containing acetic acid and exhibit a shorter adaptation period to acetic acid (Haitani et al., 2012). Global gene transcription was investigated, and upregulations of *HAA1* and *AFT1* were observed. *AFT1* is a transcription factor involved in iron-regulatory mechanisms and is activated by exposure to weak organic acids, and the transcription factor *HAA1* is involved in the adaptation to weak acid stress. Engineering of *HAA1* for improved tolerance toward acetic acid is discussed below.

### Transcription Machinery Engineering

Overexpression of *HAA1* allowed robust *S. cerevisiae* growth and sugar consumption in the presence of 4 g/l acetic acid (Cunha et al., 2018). The strain overexpressing *HAA1* also exhibited a more robust cell wall after exposure to acetic acid stress and higher resistance to lyticase, an enzyme that can digest the yeast cell wall. In another study, global transcription machinery engineering performed on *HAA1* allowed the identification of a point mutation S135F, which conferred higher tolerance toward acetic acid (Swinnen et al., 2017). The level of acetic acid tolerance obtained by this mutant was similar to that obtained by *HAA1* overexpression, indicating that the mutated *HAA1* was more active than the wild-type *HAA1* and the enhanced tolerance was not due to an increase in *HAA1* transcript level.

In *S. cerevisiae, MIG1* encodes a catabolite repressor which can repress the expression of several genes for the metabolism of acetate under high glucose concentrations (Balderas-Hernandez et al., 2018). *MIG1* is also involved in the glucose repression of genes related to stress tolerance, such as the salt stress-inducible P-type ATPase sodium pump ENA1p. The deletion of *MIG1* was shown to enable *S. cerevisiae* to tolerate the lethal concentrations of 5 g/l of acetic acid and 1.75 g/l of formic acid without significantly affecting the fermentative production of ethanol. In the presence of 5 g/l of acetic acid in glucose-containing media, Δ*mig1* mutant was able to produce 4.06 g/l of ethanol, whereas the wild-type *S. cerevisiae* could produce 4.50 g/l of ethanol in media without acetic acid.

### Adaptive Laboratory Evolution

ALE was performed to improve the tolerance of *S. cerevisiae* toward two aromatic acids, coumaric acid and ferulic acid (Pereira et al., 2020). *S. cerevisiae* was serially transferred into fresh media containing the aromatic acids with concentrations increasing from 0.2 g/l to the maximum solubility of 1 g/l at a constant pH of 3.5. The evolved strains obtained could tolerate 1 g/l of the aromatic acids and had a significantly higher growth rate than the wild-type strain. Whole genome sequencing revealed point mutations in the transcriptional activator *ARO80*. Overexpression of *ESBP6*, a gene similar to mammalian monocarboxylate permease and regulated by *ARO80*, was also found to improve the tolerance to aromatic acids by secreting them out of the cells.

In another study, *S. cerevisiae* was subjected to serial passage into fresh media with increasing concentrations of dicarboxylic acids: glutaric acid, adipic acid, and pimelic acid (Pereira et al., 2019). Several clones from the evolved population were screened in the presence of inhibiting concentrations of dicarboxylic acids, and some of the best performing clones were identified. An increase in the read coverage of a region in chromosome II containing *QDR3* was revealed through whole genome sequencing. QDR3 is a multidrug transporter which can confer tolerance to some drugs, such as quinidine and bleomycin. Overexpression of *QDR3* in wild-type *S. cerevisiae* was shown to be sufficient to confer tolerance to all three dicarboxylic acids tested as well as muconic acid and glutaconic acid.

*E. coli* strain ML115 was evolved by serial transfer in media with increasing concentrations of octanoic acid (Royce et al., 2015). The evolved strain was able to tolerate high concentrations of octanoic acid, hexanoic acid, and decanoic acid, as well as alcohols, such as n-butanol and isobutanol. Octanoic acid production by the final strain was 5.63-fold higher than that by the parent strain. The evolved strain also had a lower saturated: unsaturated ratio, higher membrane polarization, and more significant perturbations to membrane content than the parent strain.

ALE was also performed to improve the tolerance of *Pichia kudriavzevii* NG7 to lactic acid (Park et al., 2018). The final strain was able to tolerate a lactic acid concentration of 6% and produce twofold higher of lactic acid than the parent strain. Mutation mining revealed an in-frame deletion in *PAR1*, a potential transcriptional regulator with unclear functions. Reverse engineering by knocking out *PAR1* in the parent strain or restoration of *PAR1* in the evolved strain verified the *PAR1* deletion was responsible for the improved tolerance to lactic acid.

### Genome-Scale Engineering

A yeast genomic library was transformed into *S. cerevisiae* CEN.PK2-1D, and the fast-growing transformants were selected on synthetic complete medium agar plates containing 35 g/l of glucose and 3 g/l of acetic acid (Oh et al., 2019). The best performer contained a plasmid for overexpression of *RCK1*, a gene encoding a protein kinase involved in oxidative stress. Under the presence of acetic acid, the RCK1-overexpressing strain exhibited a twofold higher ethanol productivity than the control strain in glucose fermentation. Furthermore, overexpression of *RCK1* lowered intracellular ROS levels by 40% and thus reduced the oxidative stress caused by acetic acid.

Genome shuffling is another genome-scale engineering strategy which employs recombination processes to facilitate the evolution of strains with improved functions (Hu et al., 2018). Using N-methyl-N'-nitro-N-nitrosoguanidine and UV treatments to screen for starter strains which could grow at pH 4.8 followed by genome shuffling, an *A. succinogenes* mutant with robust growth under a pH of 3.5 was isolated. This resulting mutant could produce 2.6-fold higher succinic acid in shake flask fermentation than the original strain.

A library of 345 nonessential histone point mutants were screened using agar plates for sensitivity and resistance of *S. cerevisiae* toward acetic acid. Six histone H3/H4 acetic acid-resistant and 18 histone H3/H4 acetic acid-sensitive mutants were identified (Liu et al., 2014). In the presence of 60 mM of acetic acid, these histone mutants showed shorter lag time and faster ethanol production than the parent strain. Two acetic acid-resistant mutants H3 K37A and H4 K16Q were further subjected to genome-wide transcriptome analysis, and upregulations of genes related to energy production and antioxidative stress were observed. This was the first study describing the effects of histone H3/H4 mutations on acetic acid tolerance.

## Engineering for Tolerance to Cellulosic Inhibitors

### Transcriptome Analysis-Guided Engineering

*Corynebacterium glutamicum* S9114 can tolerate aldehyde inhibitors including furfural, HMF, 4-hydroxybenzaldehyde, vanillin, and syringaldehyde by converting those into the less toxic alcohols or acids (Zhou et al., 2019). Transcriptional analysis of 93 genes responsible for the aldehyde conversion was performed. One gene encoding an alcohol dehydrogenase was observed to be overexpressed in response to exposure to all five aldehyde inhibitors. Overexpression of this gene in *C. glutamicum* S9114 further improved the conversion ratio of five aldehydes and enhanced the cell growths to a certain extent.

### Transcription Machinery Engineering

The growth rates of 30 *S. cerevisiae* mutants with deletions in transcription factors involved in multidrug resistance in simple and complex inhibition scenarios, including coniferyl aldehyde, furfural, HMF, sugarcane bagasse pretreatment liquid, and spruce pretreatment liquid, were determined (Wu et al., 2017). Deletion mutants of 8 transcription factors (*STB5, YAP1, WAR1, RPN4, CAT8, PDR8, PUT3*, and *GZF3*) had significantly lower growth rates than the control in the presence of lignocellulosic inhibitors. Overexpression of these transcription factors in a wild-type *S. cerevisiae* lab strain rendered it more tolerant to inhibitors. Nevertheless, in another study, overexpression of these transcription factors in some *S. cerevisiae* environmental isolates failed to enhance inhibitor tolerance, which might be due to the existing robustness and tolerance of these strains (Mertens et al., 2021).

To enhance the furfural tolerance in *Zymomonas mobilis*, a library of the global transcription sigma factor *RpoD* mutants were generated using error-prone PCR, and the best-performing mutant strains were selected using 3 g/l furfural (Tan et al., 2015). The best mutant strain could reach the maximum cell density 1.3 times faster than the parent strain in the presence of furfural. This mutant strain also consumed glucose faster and produced more ethanol in fermentation under furfural stress.

Exposure of *S. cerevisiae* to HMF and furfural was found to induce the accumulation of ROS, leading to the damage to several cellular organelles (Kim & Hahn, 2013). HMF and furfural were also found to be thiol-reactive electrophiles that activated *YAP1*, a gene encoding a basic leucine zipper transcription factor necessary for oxidative stress tolerance. Overexpression of an active mutant of *YAP1* upregulated genes encoding antioxidant enzymes, such as *CTA1* and *GLR1*, and genes involved in the reduction of furan aldehydes, such as *ADH7* and *GRE2*, leading to increased tolerance of *S. cerevisiae* to furfural and HMF.

### Adaptive Laboratory Evolution

ALE was performed to improve the inhibitor tolerance of *C. glutamicum* S9114 by serial transfer into corn stover hydrolysate containing inhibitors (Wang et al., 2018a). The evolved strain exhibited faster conversion of toxic aldehyde inhibitors to the corresponding alcohols or acids. This strain was also able to produce 68.4% more glutamic acid in corn stover hydrolysate compared to the parent strain. Whole genome sequencing and transcriptional analysis revealed that the phosphoenolpyruvate: sugar phosphotransferase system transport and the pentose phosphate pathway were significantly upregulated, which could lead to more available NADPH for the aldehyde conversion and thus higher tolerance.

ALE was performed to evolve *Yarrowia lipolytica* to tolerate a high concentration of ferulic acid, one of the main phenolics in lignocellulosic hydrolysate (Wang et al., 2021b). The final strain could tolerate 1.5 g/l of ferulic acid, whereas a concentration of 0.5 g/l was lethal enough for the parent strain. Transcriptome analysis was then performed to identify upregulations of four genes (two multidrug resistance-associated proteins and two integral membrane proteins) involved in drug resistance and membrane integrity. Overexpression of these genes in the parent strain rendered it more tolerant to ferulic acid as well as vanillic acid.

ALE was also conducted to improve the tolerance of an ethanologenic *E. coli* LY180 to furfural (Miller et al., 2009). After 53 serial transfers in pH-controlled bioreactors in xylose-containing medium with furfural with concentrations increasing from 0.5 g/l to 1.3 g/l, a resulting strain EMFR9 was obtained. This evolved strain, in the presence of 1 g/l furfural, could grow at rate similar to the parent strain without furfural. mRNA analysis determined that the silencing of two NADPH-dependent oxidoreductase genes, *yqhD* and *dkgA*, accounted for the improved tolerance to furfural. Surprisingly, both *yqhD* and *dkgA* were found to possess NADPH-dependent furfural reductase activities, which convert furfural to the less toxic furfuryl alcohol. Both genes were also determined to have low *K_m_* values for NADPH, and thus the growth inhibition by furfural was likely because of the competition for NADPH between *yqhD* and *dkgA* and essential NADPH-dependent biosynthetic reactions.

### Genome-Scale Engineering

**Fig. 3.**
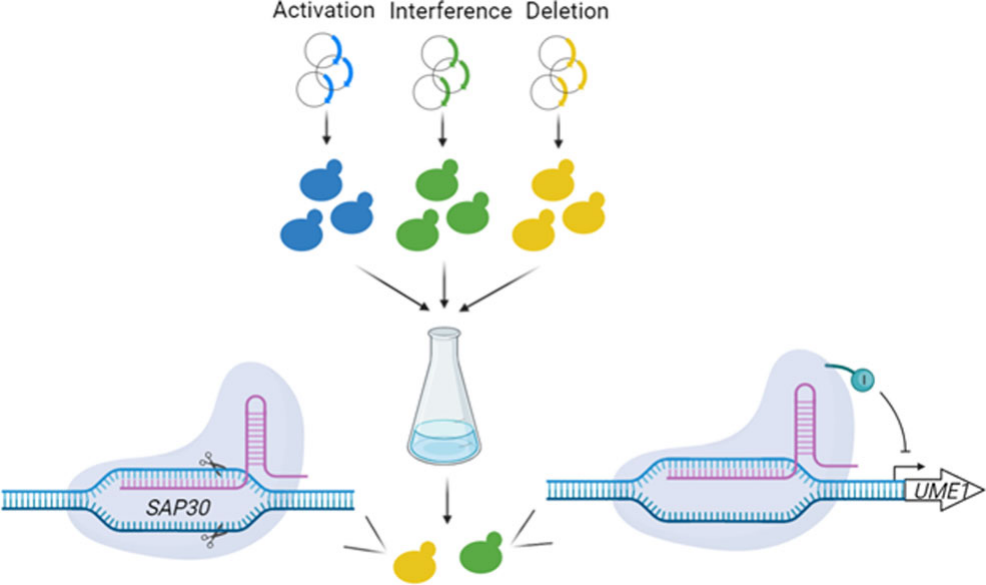
Scheme for MAGIC (Lian et al., 2019). Pooled libraries for activation, interference, and deletion were transformed into *S. cerevisiae* harboring the CRISPR-AID system. The transformants were subjected to growth enrichment under 5 mM furfural, and *SAP30* deletion and *UME1* interference were among the highly enriched guides. Reverse engineering verified these genetic modifications enabled *S. cerevisiae* tolerance to 5 mM furfural.

Genome-scale engineering approaches have been developed and applied to improve the tolerance of *S. cerevisiae* to lignocellulosic inhibitors (Ding et al., 2020). RAGE (RNAi-assisted genome evolution) allows the accumulation of beneficial gene downregulations to achieve the desired phenotypes (Si et al., 2015a; Si et al., 2017). CHAnGE (CRISPR–Cas9-assisted and homology-directed-repair-assisted genome-scale engineering) can quickly construct a library of genome-wide deletions, disrupting 98% of target sequences with editing efficiency of 82%, whereas MAGIC (multifunctional genome-wide CRISPR) combines the trifunctional CRISPR-AID (activation, interference, and deletion) system with array-synthesized oligo pools to generate libraries of deletion, activation, and interference (Bao et al., 2018; Lian et al., 2019). These methods allowed the identification of novel genetic modifications that can mitigate stress from lignocellulosic inhibitors on *S. cerevisiae* growth. For example, using MAGIC, interference of *UME1*, a gene encoding a component of the Rpd3L histone deacetylase complex, was found to allow *S. cerevisiae* to grow in the presence of 5 mM furfural, suggesting histone modification might play a role in tolerance to furfural (Fig. 3). Interestingly, downregulation of *SIZ1*, an E3 SUMO-protein ligase, identified by RAGE and MAGIC and *SIZ1* deletion determined by CHAnGE were found to improve furfural tolerance.

## Insights on Engineering a Robust Microorganism for Organic Acid Production

The construction of robust microorganisms with high tolerance to low pH and acid stress is important for efficient microbial productions of organic acids and has been attained using transcriptome analysis-guided engineering, engineering of transcription machinery, ALE, and genome-scale engineering approaches. Nevertheless, regardless of which method was utilized, the acquired genetic modifications usually share some common mechanisms and insights. Strains with improved low pH tolerance can have higher-energy metabolism to increase the intracellular ATP supply, which is necessary for ATP-driven proton pumps to export protons out of the cells (Liu et al., 2015). For example, genes related to energy production were found to be upregulated in the evolved *E. coli* strain with improved growth rate at pH 5.5 or in the acetic acid-resistant *S. cerevisiae* histone mutants (Du et al., 2020; Liu et al., 2014). Furthermore, engineered strains can confer acid tolerance by changing the fluidity and rigidity of the cell membrane and cell wall, which become less permeable to organic acids. Alterations in cell membrane were observed in the *E. coli* strain adapted to high concentration of octanoic acid (Royce et al., 2015), and genes mapping in the integral component of membrane of gene ontology terms were found to be highly upregulated in the *Y. lipolytica* strain adapted to lethal concentration of ferulic acid (Wang et al., 2021b). Overexpression of genes involved in the conversion of organic acids and lignocellulosic inhibitors into less toxic compounds also accounts for the enhanced robustness of microorganisms. For instance, upregulation of CYB2, which converts lactate to pyruvate, improved the tolerance of *S. cerevisiae* toward lactic acid (Fletcher et al., 2017), and overexpression of an alcohol dehydrogenase in *C. glutamicum* S9114 alleviated the toxicity of aldehyde lignocellulosic inhibitors by converting them to the corresponding alcohols or acids (Zhou et al., 2019). Overall, the acquired mechanisms in strains with improved tolerance to low pH, acid stress, and lignocellulosic inhibitors are typically associated with energy metabolism, cell wall synthesis, proton and acid secretion, and product degradation. Genes related to these processes can potentially serve as rational targets for improving the robustness of microorganisms and future metabolic engineering of microorganisms for organic acid productions.

Although transcriptome analysis-guided engineering, transcription machinery engineering, ALE, and genome-scale engineering have been applied to improve host robustness with high success rates, these strategies still have some limitations. While transcriptome analysis enables the prediction of targets for strain development, phenotypic responses may not always be directly related to the transcriptional patterns (Jewett et al., 2005). Transcription factors not only regulate genes related to stress tolerance but also genes involved in essential metabolic functions; modifications of transcription factors can lead to reduced fitness and may not be desired (Wang et al., 2021a). Furthermore, ALE does not necessitate *a priori* knowledge of the mechanisms of acid tolerance and can be performed without using any genetic tools, but ALE can be very arduous and requires days or even months of repetitive manual transferring and adaptation (Wu et al., 2021). Finally, genome-scale engineering methods can create large mutant libraries and enrich the mutants with high acid tolerance. However, some CRISPR-based tools involve double-stranded DNA breaks, which can be toxic to microorganisms, and have potential off-target effects (Lian et al., 2018a).

## Conclusion and Future Perspectives

Microbial organic acid production faces the challenges imposed by the poor tolerance of microorganisms to low pH and acid stress. To achieve economic and efficient fermentation processes for production of organic acids, it is crucial to enhance the robustness of microbial cell factories. Transcriptome analysis-guided engineering, transcription machinery engineering, ALE, and genome-scale engineering approaches have been successfully applied to improve the acid tolerance of various microorganisms. Furthermore, microorganisms have developed several mechanisms to survive in highly acidic environments, such as pH homeostasis, cell membrane modifications, and regulation of energy metabolism (Guan & Liu, 2020). Although the mechanisms for responding to acid stress might be similar, some microbes are still better than others at coping with and adapting to acid stress. Advances in systems and synthetic biology might allow us to develop a more comprehensive understanding of the tolerance mechanisms of the microorganisms with outstanding resistance to low pH and acid stress. For example, the nonconventional yeast *I. orientalis* is well known for its exceptional resistance to low pH and high concentrations of organic acids (Han et al., 2014); several fundamental genetic tools have been developed for this species, paving the way for the elucidation of its mechanism for acid stress tolerance (Cao et al., 2020a; Tran et al., 2019). Transferring of these superior tolerance mechanisms to existing microbial cell factories with poor tolerance to acid stress may improve their performance in production of organic acids. Finally, although genome-scale engineering tools such as MAGIC and CHAnGE have been only applied to improve tolerance of *S. cerevisiae* to lignocellulosic inhibitors (Bao et al., 2018; Lian et al., 2019), they are particularly helpful to select for growth-associated phenotypes, such as tolerance to highly acidic conditions and high concentrations of organic acids. These genome-scale engineering tools can construct mutant libraries covering the entire genome, and growth enrichment under low pH or acid stresses can enrich the mutants with high tolerance toward these stresses. Furthermore, these genome-scale engineering approaches can be performed in a timescale much shorter than ALE, and the genetic modifications resulting in improved tolerance can be quickly identified by sequencing of the plasmids harbored in the enriched mutants (Cao et al., 2020b).

## Data Availability

Data sharing is not applicable to this article as no new data were obtained or analyzed in this review.
